# New Polymers for Needleless Electrospinning from Low-Toxic Solvents

**DOI:** 10.3390/nano9010052

**Published:** 2019-01-02

**Authors:** Martin Wortmann, Natalie Frese, Lilia Sabantina, Richard Petkau, Franziska Kinzel, Armin Gölzhäuser, Elmar Moritzer, Bruno Hüsgen, Andrea Ehrmann

**Affiliations:** 1 Faculty of Engineering and Mathematics, Bielefeld University of Applied Sciences, 33619 Bielefeld, Germany; lilia.sabantina@fh-bielefeld.de (L.S.); r.petkau95@googlemail.com (R.P.); franziska.kinzel@fh-bielefeld.de (F.K.); bruno.huesgen@fh-bielefeld.de (B.H.); andrea.ehrmann@fh-bielefeld.de (A.E.); 2 Faculty of Physics, Bielefeld University, 33615 Bielefeld, Germany; nfrese@physik.uni-bielefeld.de (N.F.); ag@uni-bielefeld.de (A.G.); 3 Faculty of Mechanical Engineering, Paderborn University, 33098 Paderborn, Germany; elmar.moritzer@ktp.upb.de

**Keywords:** electrospinning, nanofiber, polymer solution, acetone, DMSO, water-soluble polymers

## Abstract

Electrospinning is a new technology whose scope is gradually being developed. For this reason, the number of known polymer–solvent combinations for electrospinning is still very low despite the enormous variety of substances that are potentially available. In particular, electrospinning from low-toxic solvents, such as the use of dimethyl sulfoxide (DMSO) in medical technology, is rare in the relevant scientific literature. Therefore, we present in this work a series of new polymers that are applicable for electrospinning from DMSO. From a wide range of synthetic polymers tested, poly(vinyl alcohol) (PVOH), poly(2ethyl2oxazolene) (PEOZ), and poly(vinylpyrrolidone) (PVP) as water-soluble polymers and poly(styrene-co-acrylonitrile) (SAN), poly(vinyl alcohol-co-ethylene) (EVOH), and acrylonitrile butadiene styrene (ABS) as water-insoluble polymers were found to be suitable for the production of nanofibers. Furthermore, the influence of acetone as a volatile solvent additive in DMSO on the fiber morphology of these polymers was investigated. Analyses of the fiber morphology by helium ion microscopy (HIM) showed significantly different fiber diameters for different polymers and a reduction in beads and branches with increasing acetone content.

## 1. Introduction

Electrospinning can be used to produce fine fibers with diameters between some 10 nanometers and a few microns [1,2]. Traditionally, electrospinning has been performed using a syringe that presses a molten or dissolved polymer through a needle; a high electric field then drags the polymer solution to a substrate on which it is placed. Needleless technologies, however, are of high interest as they allow processes to be upscaled to an industrial scale [3] and sometimes enable introducing special properties of the resulting nanofiber mats, such as a high orientation of the fibers [4,5].

Only few polymers, however, can be electrospun from aqueous solutions or low-toxic solvents. A typical example of a water-soluble polymer is poly(ethylene oxide) (PEO) [6,7], which can also be used as a spinning agent for a number of other (bio-)polymers that are not electrospinnable solely [8,9]. Amongst the water-resistant polymers, polyacrylonitrile (PAN), which can be dissolved in dimethyl sulfoxide (DMSO), is often used [10,11]. PAN is of special interest because it can be used as a precursor for the production of carbon nanofibers [12,13]. Compared to many other solvents, DMSO has a reportedly low toxicity, which may depend upon the concentration. However, there have been reported cases of harmful effects from DMSO [14] and, as with all chemicals, appropriate precautions should be taken. In this work, DMSO was specifically chosen not only for its low toxicity but because it dissolves a great number of amorphous polymers, even compared to acetone, making DMSO a versatile solvent for verifying the spinnability of new polymers for the electrospinning process. Many polymers that are soluble in other nontoxic solvents, such as water or ethanol, can also be dissolved in DMSO. Ethanol, on the other hand, cannot be used if it is not very diluted due to the risk of explosion in wire-based electrospinning.

Electrospinning from DMSO is of special importance for the development of materials for applications in biotechnology and medical applications, enabling cell growth without the possible problem of undesired, toxic solvent residues. While Fourier transform infrared spectroscopy (FTIR) investigations on PAN or PAN/gelatin nanofiber mats electrospun from DMSO have shown no measurable remains of DMSO in the nanofiber mats directly after electrospinning [15,16,17], tests in a biotechnological application [18] revealed smallest residues of acetic acid in a polyamide 6 (PA6) nanofiber mat electrospun from acetic/formic acid. This underlines the importance of using low-toxic solvents to reduce the possible influence on cells growing on such electrospun nanofiber mats as much as possible.

Tests on electrospinning polymers from DMSO, however, are reported scarcely. It should be mentioned that using DMSO to dissolve water-soluble polymers can also be of technological interest for blends of the respective polymers with nanoparticles that are oxidized in aqueous solutions, such as several magnetic nanoparticles [19]. Recently, investigations were performed using DMSO for electrospinning biopolymers, such as collagen [20] or starch [21]. Another polymer that has recently been shown to be spinnable from DMSO is poly(ethylene-co-vinyl alcohol) (EVOH) [22,23].

Other polymers, such as poly(vinyl alcohol) (PVOH), poly(2ethyl2oxazolene) (PEOZ), poly(vinylpyrrolidone) (PVP), poly(styrene-co-acrylonitrile) (SAN), or acrylonitrile butadiene styrene (ABS), have not yet reported to be spinnable from DMSO. However, there have been studies on the spinnability of ABS from dimethylformamide [24], PVP from dichloromethane [25], PVOH from water [26], PEOZ from water, dimethylformamide and tetrahydrofurane [27], and SAN from 1,2-dichloethane [28].

Here, we report on the first electrospinning experiments on these polymers, indicating that most of them can be electrospun from DMSO solutions as a low-toxic alternative to the aforementioned solvents or water.

Furthermore, the influence of acetone on the DMSO solutions was tested. The influence of binary solvents on the fiber morphology has been reported in several studies, such as References [29,30], while acetone as the volatile solvent component in particular has not yet been studied with the polymers investigated here.

## 2. Materials and Methods

For the nanofiber mats presented in this work, different polymers (Table 1) were dissolved to different concentrations in DMSO (minimum 99.9%, purchased from S3 Chemicals, Bad Oeynhausen, Germany).

The concentrations in the respective polymer solutions were determined in extensive preliminary investigations. For this, the concentration was first roughly chosen for all polymers so that the viscosity, according to experience, is suitable for electrospinning. The concentrations were chosen so that the viscosities of all solutions were approximately equal. The exact concentration used here was again optimized in further experiments. The polymers were present either as granules or as a powder and, depending on the polymer, were dissolved between room temperature and 70 °C on a magnetic stirrer. Acetone of 0, 10, and 20 wt% were added to each solution as the solvent content. The structural formulas of the polymers used are shown in Table 2.

Electrospinning was performed using the wire-based electrospinning machine Nanospider Lab (Elmarco, Liberec, Czech Republic). Depending on the polymer used, a voltage between 60 and 80 kV and a carriage speed between 50 and 150 mm/s was applied. Nozzle diameter of 0.8 mm, spinning time of 20 min, and distance between the electrode wires of 240 mm were kept the same for all experiments. The relative humidity in the spinning chamber was 34%, and the temperature during spinning was 23 °C.

Surface free energy measurements were performed with distilled water, ethylene glycol, and diiodomethane at 22 °C using the contact angle measurement device OCA 15 Pro (DataPhysics Instruments, Filderstadt, Germany). The surface free energy was calculated according to the Owens-Wendt-Rabel-Kaelble (OWRK) method [31].

**Figure 1:**
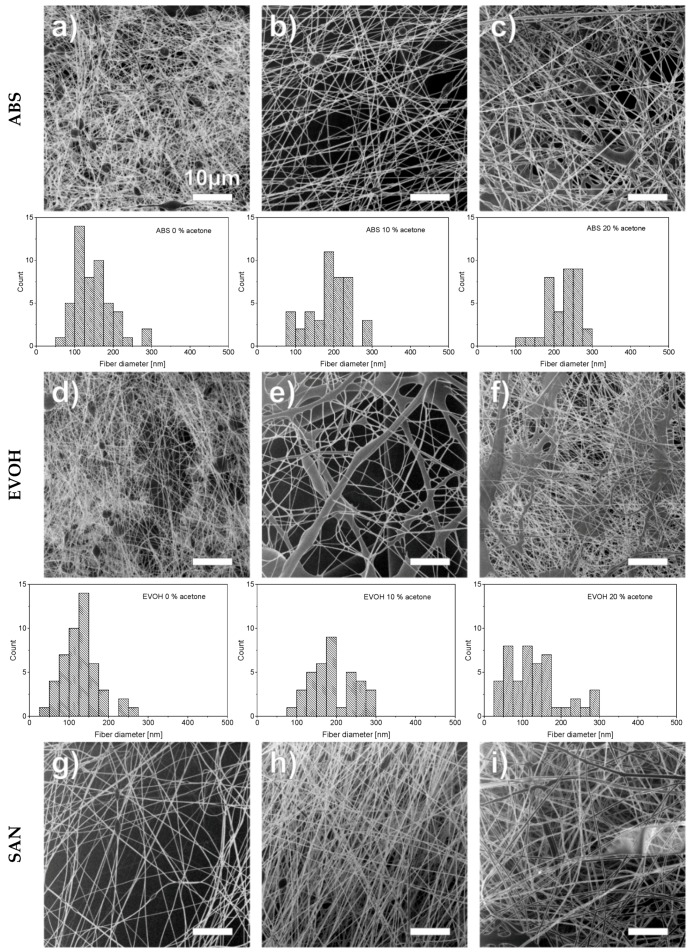

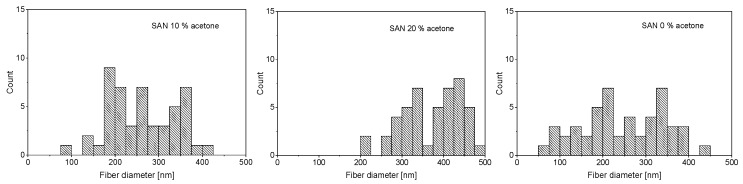
Helium ion microscope (HIM) images of acrylonitrile butadiene styrene (ABS), poly(vinyl alcohol-co-ethylene) (EVOH), and poly(styrene-co-acrylonitrile) (SAN) nanofibers spun from different DSMO:acetone weight proportion of (**a**,**d**,**g**) 100:0, (**b**,**e**,**h**) 90:10, and (**c**,**f**,**i**) 80:20, with respective histograms of fiber diameter distributions.

**Figure 2:**
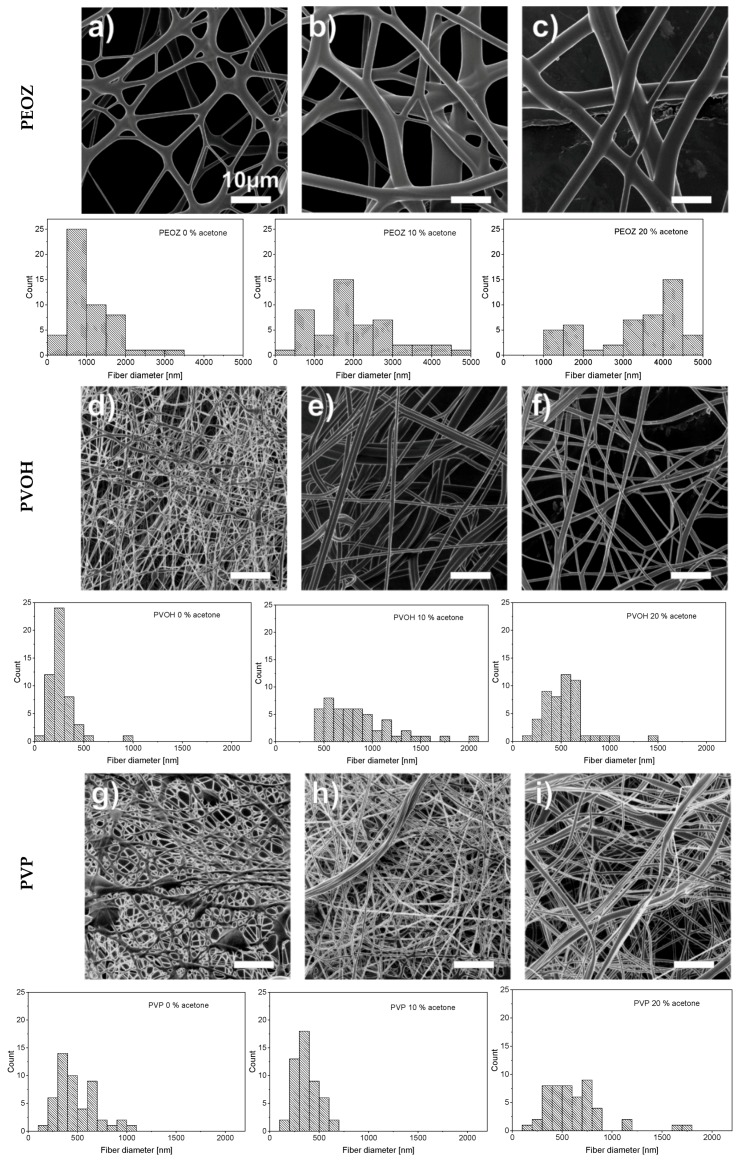
HIM images of poly(2ethyl2oxazolene) (PEOZ), poly(vinyl alcohol) (PVOH), and poly(vinylpyrrolidone) (PVP) nanofibers spun from different DSMO:acetone weight proportion of (**a**,**d**,**g**) 100:0, (**b**,**e**,**h**) 90:10, and (**c**,**f**,**i**) 80:20, with respective histograms of fiber diameter distributions.

**Figure 3:**
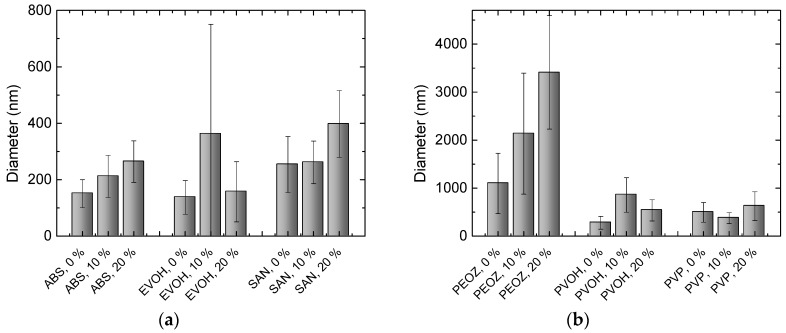
Average diameters of nanofibers electrospun (**a**) from different water-insoluble polymers; (**b**) from different water-soluble polymers.

Helium ion microscopy pictures were taken with an Orion Plus (Carl Zeiss, Jena, Germany) at an acceleration voltage of 35 kV and a current of 0.4–0.5 pA. An electron flood gun was used to avoid charging effects during the secondary electron detection. The software ImageJ 1.51j8 (from National Institutes of Health, Bethesda, MD, USA) was applied to determine the nanofiber diameters from 50 fibers per sample. Individual outliers are not shown in Figure 1 and Figure 2 for the sake of clarity but are taken into account in the overview diagrams in Figure 3.

## 3. Results and Discussion

This work deals with the polymers listed in Table 1, which were found to be applicable for electrospinning in extensive preliminary investigations. The polymers that were not considered further after the preliminary investigations, on the other hand, will only be briefly discussed here.

The investigated polymers that could not be easily electrospun from DMSO were acrylonitrile styrene acrylate (ASA), polyamide (PA, amorphous copolyamide from ε-caprolactam, hexane-1,6-diamine, hexanedioic acid, and 4,4-diaminodicyclohexylmethane), polycarbonate (PC, based on bisphenol A and phosgene), poly(vinyl chloride) (PVC), and poly(methyl methacrylate) (PMMA). This is not to say that these polymers are fundamentally not applicable for electrospinning from DMSO. It has been found that some of the tested polymers, such as PVC or PA, although soluble at higher temperatures, thicken or jelly at room temperature. A heated solvent container, for example, could solve this problem. It is also possible that the specific brands used here are not soluble in sufficiently high concentrations to be electrospun. As has already been shown by various publications, the spinnability and the morphology of the fibers depend strongly on the molecular weight and the solvent used. As the name of the polymers does not in itself say anything about properties such as molecular weight distribution, crystallinity (even most amorphous polymers have some degree of crystallinity), degree of branching and crosslinking, or moisture content, it is quite possible that the polymers mentioned here are applicable under other conditions. However, extensive investigations would be required for each individual polymer to determine this, and as this work focuses on providing a rough overview of new, applicable polymers to encourage new studies in the field, this will not be discussed any further.

As the polarity of the polymer to be spun is relevant to both solubility and electrospinning, the applicable polymers were examined by contact angle measurement to determine the surface free energy. For this purpose, the polymers were either pressed under slight pressure above their glass transition temperature to plates or processed to thin films by means of volatile solvents, such as acetone or ethanol. The measured surface free energies of the bulk samples are shown in Table 3.

The measured values are essentially consistent with literature values for these or similar polymers. What is common to all polymers used here is their high polar content of surface free energy. As the polarity of the polymers determines the orientation in the electric field, it is not surprising that polar polymers are the most suitable for use in the electrospinning process. The values measured here are rather high compared to most other industrially used polymers. 

As water with a value of 63.1 kcal/mol has the highest polarity on the *E_T_*(30) scale (based on the negative solvatochromic Pyridinium-N-phenolate betaine-30 dye), it corresponds to the expectation that the water-soluble polymers (PVOH, PEOZ, and PVP) have the highest polar content of the surface free energy. Acetone (42.2 kcal/mol) and DMSO (45.1 kcal/mol) are miscible due to similar polarity, with most of the polymers tested here not being soluble in pure acetone but only in the binary solvent mixture [33]. As shown in Reference [30], in binary solvent mixtures, high differences in volatility and vapor pressure among the mixed solvents can induce phase separation in electrospinning, leading to highly porous fiber topologies [34]. High-resolution imaging of the fiber topology necessary to test this hypnosis was not possible in this work because the helium ion beam of the helium ion microscope (HIM) (as opposed to electron beams in a scanning electron microscope (SEM)) can move individual fibers during imaging.

Figure 1 shows the fibers of water-insoluble polymers with different proportions of acetone and the associated fiber diameters in the respective histograms. As only small areas of the nanofiber mats are visible at this high magnification, care has been taken to depict representative areas of the nanofiber mats, including a representative distribution of fiber diameters, especially for the cases where very large differences were found between the thinnest and the thickest fibers (e.g., Figure 1e). The magnification level used is a compromise between the possibility of analyzing the fiber morphology and ensuring a good overview of fiber density and homogeneity. 

The morphology of the water-insoluble polymers, i.e., ABS, EVOH, and SAN, turned out to be much more difficult to characterize compared to the water-soluble polymers, i.e., PEOZ, PVOH, and PVP. The reason for this is because it has been found that the resulting nanofiber mats are very inhomogeneous in small scales, which makes it difficult to genuinely generate representative mappings. A potential reason for this could be a broader molecular weight distribution of the polymers, which could not be further investigated in this work. To address this issue, further investigations of the process parameters and solution compositions will be necessary. 

However, what could be observed in principle is that, with increasing acetone content in all water-insoluble polymers, the number of bead-like thickening in the fibers (dark round areas, e.g., in Figure 1a,b,d,g) decreased. The reason for this is the higher volatility due to the acetone. As discussed in References [30,35,36], the transition from grain-like or branched structures to straight fibers depends on a critical minimum polymer concentration, *c*_e_. Above *c*_e_, a further increase in polymer concentration leads to a decrease in the number of beads and an increase in fiber diameter. The bead count reduction at constant polymer concentration, as shown in Figure 1c,f,i, suggests that *c*_e_ can be reduced by the addition of acetone.

The fibers of water-soluble polymers with different proportions of acetone and the associated fiber diameter histograms are shown in Figure 2. While the addition of acetone to the water-insoluble polymers led to a decrease in bead formation, a transition from a rather network-like structure to straight, defined fibers could be observed for the water-soluble polymers. Comparing Figure 2a,d,g to Figure 2c,f,i, it can be clearly seen that, in all water-soluble polymers, the number of cross-links of individual fibers decreased with increasing acetone content. As less solvent reaches the support material due to the higher volatility of the acetone, fewer bonds are formed between the fibers. For this reason, the PEOZ, PVOH, and PVP solutions without acetone, as seen in Figure 2a,d,g, showed significantly more cross-linking points. The same effect means that thickening by solvent residues can be reduced or completely prevented with increasing acetone content. As can be seen in Figure 2a,g in particular, there was a clear transition from a more network-like structure to separated fibers in PEOZ and PVP.

As mentioned earlier, the addition of acetone leads to a potential reduction in the critical minimum concentration *c*_e_, which in turn is known to increase the fiber diameter. Furthermore, as demonstrated in Reference [36], surface tension reduction and increasing volatility results in an increase in average fiber diameter. As acetone is less polar than DMSO and the surface tension of the solution is thus reduced by the acetone supplementation, it would as well be conceivable that this effect contributed to the increase in fiber diameter. As discussed above, it has been found that fiber morphology and distribution is inhomogeneous in small scales. The broad distribution of fiber diameters, as seen in the histograms in Figure 1 and Figure 2, explains the large standard deviations in Figure 3. Although the effect is not constant for all polymers, there is a clear trend toward larger fiber diameters with increasing acetone content. Results that do not support this thesis are either within the standard deviations, such as PVP without and PVOH with 10% acetone, or can be identified as outliers, such as EVOH with 10% acetone, as seen in Figure 1e.

As has been shown in several studies, there is a strong correlation between the average fiber diameter and polymer concentration. Generally, it was found that the water-insoluble polymers, as seen in Figure 3a, had a significantly lower average fiber diameter than the water-soluble polymers, as seen in Figure 3b. This is likely attributed to the tendency toward higher polymer concentrations in the water-soluble polymers (see Table 1). As can be seen in Figure 2a–c and Figure 3b, PEOZ showed by far the largest average fiber diameters, which can be explained by the fact that the PEOZ solution had, with 40%, by far the highest polymer concentration to achieve a suitable viscosity.

## 4. Conclusions

From a variety of tested polymers, several polymers that can be spun from DMSO have been presented in this paper. Some of these polymers have already been tested in other studies for the production of nanofibers from toxic solvents. Finding new polymers or new solvents for known polymers opens up many possibilities for further studies. The solvent not only has a significant influence on spinnability and fiber morphology but also has the potential for future application in medicine and food technology.

Moreover, it has been found that the critical minimum concentration, *c*_e_, of a polymer solution for producing nanofibers can be reduced by the addition of a volatile solvent, resulting in an increased average fiber diameter. In addition, the results suggest that the supplementation of acetone has a positive effect on the homogeneity of the fiber morphology, reducing the number of beads and branches. As this effect could be observed in all polymers studied, it is quite conceivable that the addition of a volatile solvent, such as acetone, has a positive effect on nanofibers from other polymers too. A further increase in acetone, however, is not permitted, at least with the system used here, for safety reasons because the flashpoint of the solvent must not be further reduced to values near room temperature.

There are countless applications for different nanofiber materials that are currently being discussed. In particular, water-soluble nanofibers are gaining increasingly more attention. To name just a few possible applications, as demonstrated in Reference [25], nanofibers from polymer blends can be used to make highly porous nanofibers by selectively removing a component of the blend; as examined in Reference [37], amongst others, nanofibers are used as removable core templates for various nanotubes; and as described in Reference [38], they are used as oral, fast-dissolving drug delivery membranes for medical use.

In future investigations, it will be necessary to carry out detailed parameter studies for the new polymer/solvent combinations to examine the correlation between process parameters and fiber morphology. For further investigations it is planned to determine the exact *c*_e_ values of the respective polymers as well as their correlation with the acetone content.

## Figures and Tables

**Table 1:** Polymers used for electrospinning and their respective molecular weight and concentration in the polymer solution.

Abbreviation	Polymer	Supplier	M_W_ (g/mol)	Concentration (wt%)
PVOH	poly(vinyl alcohol)	Nippon Gohsei	20,000	30
PEOZ	poly(2ethyl2oxazolene)	Kremer Pigmente	200,000	40
PVP	poly(vinylpyrrolidone)	Sigma Aldrich	360,000	20
SAN	poly(styrene-co-acrylonitrile)	Sigma Aldrich	165,000	22
EVOH	poly(vinyl alcohol-co-ethylene)	Sigma Aldrich	n/a ^1^	20
ABS	acrylonitrile butadiene styrene	INEOS	n/a	25

^1^ n/a = not available.

**Table 2:** Structural formulas.

**PVOH**	**PEOZ**	**PVP**
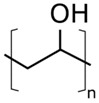	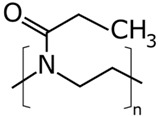	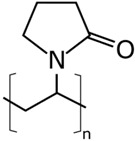
**SAN**	**ABS**	**EVOH**
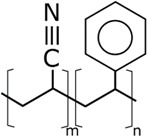	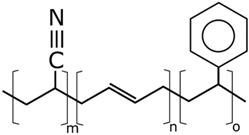	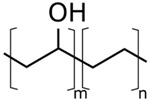

**Table 3:** Surface free energy of the bulk material.

Polymers	Total Surface Free Energy (mN/m)		Polar (mN/m)	Disperse (mN/m)
	M ^1^	SD ^2^		
PVOH *	37 [32]	n/a	n/a	n/a
PEOZ	41.86	0.80	15.21	26.65
PVP	44.72	0.81	16.25	28.47
SAN	42.82	0.76	4.07	38.75
EVOH	38.57	0.90	9.65	28.92
ABS	41.51	0.65	7.06	34.45

^1^ M = Mean, ^2^ SD = Standard Deviation; * PVOH could not be measured with the given test liquids due to the immediate interactions between the surface and the droplet.
